# Supplementary material for: Transporter modulation of molnupiravir and its metabolite β-D-N4-hydroxycytidine across the blood-brain barrier in a rat
Source: Commun Med (Lond). 2023 Oct 19;3:150. doi: 10.1038/s43856-023-00383-w (PMC10587300; doi:10.1038/s43856-023-00383-w)
Supplement: Supplementary file 1 — Supplementary Information [file 43856_2023_383_MOESM1_ESM.pdf]

Supplementary Information

Transporter modulation of molnupiravir and its metabolite β-D-N4-hydroxycytidine across the blood-brain barrier in a rat

Chun-Hao Chang[1], Wen-Ya Peng[1], Wan-Hsin Lee[1], Ling Yang[1], Tung-Yi Lin[1], Muh-Hwa Yang[2], Tung-Hu Tsai[1,3,4,5]*

[1]Institute of Traditional Medicine, School of Medicine, National Yang Ming Chiao Tung University, Taipei 112, Taiwan
[2] Institute of Clinical Medicine, National Yang Ming Chiao Tung University, Taipei, Taiwan
[3] Graduate Institute of Acupuncture Science, China Medical University, Taichung 404, Taiwan
[4] Department of Chemistry, National Sun Yat-Sen University, Kaohsiung 804, Taiwan
[5] School of Pharmacy, Kaohsiung Medical University, Kaohsiung 807, Taiwan

2. Materials and Methods

2-1. UHPLC–MS/MS conditions

A UHPLC–MS/MS system (UHPLC–MS/MS 8030, Shimadzu, Kyoto, Japan) comprising a quadrupole mass spectrometer equipped with an electrospray ionization (ESI) source was used for molnupiravir and β-d-N4-hydroxycytidine (NHC) analysis. An Acquity C-18 analytical column (50 mm × 2.1 mm, particle size 1.7 µm, Waters, MA, USA) was used to separate the analytes and dialysates. The mobile phase consisted of 2 mM ammonium acetate (adjusted to pH 4.3 by acetic acid) and acetonitrile with gradient elution at 98:2 (v/v) 2 mM ammonium acetate/acetonitrile for 0-2 min and 70:30 (v/v) 2 mM ammonium acetate/acetonitrile for 2-6 min. For 6-15 min, isocratic elution was used at 98:2 (v/v) 0.2% 2 mM ammonium acetate/acetonitrile to re-equilibrate the column, and the flow rate was 0.3 mL/min. The mobile phase was filtered through a 0.22 µm filter and degassed by an ultrasonic bath sonicator for 30 min before instrumental analysis. The injection volume of each sample was 5 µL, and the total run time was 15 min. Labsolution software (version 1.1; Shimadzu, Kyoto, Japan) was used to optimize the conditions of molnupiravir and NHC for UHPLC–MS/MS analysis. Both molnupiravir and NHC were monitored in positive ionization mode and multiple reaction monitoring (MRM) mode. The ion transitions monitored for molnupiravir and NHC were 329.0 → 128.0 m/z and 260.0 → 128.0 m/z, respectively, and the collision energies (CEs) were 15 V and 11 V, respectively (Supplementary Figure 1). NHC and molnupiravir were eluted at 0.7 and 3.7 min, respectively. The mass spectrometric conditions were as follows: ESI, positive mode; interface voltage, 4.5 kV; nebulizing gas flow, 3.0 L/min; drying gas flow, 15.0 L/min; desolvation temperature, 250 °C; heat block temperature, 400 °C; and collision-induced dissociation gas, 230 kPa.

2-2. Method Validation

The method validation was based on the bioanalytical method validation guidelines released by the US Food and Drug Administration in 2018, and the method was fully validated by linearity, precision, accuracy, stability, recovery and matrix effect tests.

The calibration curves of the blood and brain samples were generated with peak integration and nominal concentration data, and the standard solutions were diluted from the standard solution of molnupiravir (1 mg/mL). The diluted concentrations were 2.5, 5, 10, 25, 50, 100, and 250 ng/mL, which were prepared using blank dialysate. The stock solution of NHC (1 mg/mL) was diluted to 10, 50, 100, 250, 500, and 1000 ng/mL by blank dialysates. The linearity of the calibration curve was determined as a coefficient correlation (r^2) greater than 0.995 for all the standard curves.

To assess precision and accuracy, the blank blood dialysates or blank brain dialysates were spiked with molnupiravir and NHC at the lower limit of quantification (LLOQ) and low, medium and high concentrations and analyzed on the same day (intraday) or five different days (interday) with five replicates. The accuracy was determined as the similarity between the nominal concentration (C_{nom}) and observed concentration (C_{obs}), and the formula was as follows: accuracy (bias %) = $[(C_{obs}-C_{nom})/C_{nom}] \times 100$. The precision was calculated as the relative standard deviation (RSD), which means that the similarity within each C_{obs} measure to the standard deviation (S.D.) value and was obtained using the following formula (% RSD) = [standard deviation (SD)/C_{obs}] × 100%. Both the deviation values of R.S.D. (%) and bias (%) were required to be within ±15% to be considered acceptable according to the bioanalytical method validation guidelines, and an LLOQ within ±20% was acceptable.

The stability was evaluated with spiked blank blood and brain dialysates, and the evaluation included analyses of autosampler stability, bench-top stability, long-term stability and freeze–thaw stability, with three replicates of low and high concentrations. All stability determinations were compared with freshly prepared samples from the standard stock solution. Autosampler stability was evaluated by placing spiked samples at 10 °C for 6 hours; bench-top stability was determined by the stability of samples at room temperature for 6 hours; long-term stability determined using was spiked sample stability at -20 °C for more than 4 weeks; freeze–thaw stability was assessed based on the stability of samples after three freeze–thaw cycles. The sample was frozen and thawed for at least 12 hr during each cycle. To calculate stability, the formula was as follows: Stability (%) = (the peak integration of processed sample/the peak integration of the freshly prepared sample) × 100.

The in vitro recovery of the microdialysis probe was determined by measuring the concentration of

molnupiravir and NHC through the semipermeable dialysis membrane. The microdialysis probes were put into anticoagulant citrate dextrose (ACD) solution spiked with the standard solution in 1.5 mL Eppendorf tubes containing molnupiravir at concentrations of 250, 500 and 1000 ng/mL and NHC at concentrations of 500, 1000 and 2000 ng/mL. The microdialysis recovery (R_{dial}) was calculated by comparing the concentration in the dialysate (C_{dial}) with the C_{nom} of three replicates: R_{dail} (%) = (C_{dial}/C_{nom}) × 100.

Two sets of samples were prepared to assess the matrix effect during the bioanalytical method validation.

Set 1. The samples were prepared at target concentrations of 5 and 250 ng/mL for molnupiravir and concentrations of 25 and 1000 ng/mL for NHC in the mobile phase. After mixing, the solutions were transferred from Eppendorf tubes to autosampler vial inserts, and 5 μL was injected into the UHPLC–MS/MS system.

Set 2. The samples were prepared by spiking 50 μL of the appropriate concentrations of molnupiravir and NHC standard solutions. The total volume was 50 μL of dialysate collected from microdialysis, and the target concentrations were 5 and 250 ng/mL molnupiravir and 25 and 1000 ng/mL NHC. After mixing, the solutions were transferred from Eppendorf tubes into autosampler vial inserts, and 5 μL was injected into the UHPLC–MS/MS system.

By comparing the peak area integration of set 1 and set 2, the associated ion suppression or enhancement was assessed.

3. Results

3-1. Method validation

The calibration curve of molnupiravir and NHC showed good linearity (r^2) in the UHPLC–MS/MS analysis range, and both r^2 values were higher than 0.995. The LLOQ was determined when the signal-to-noise ratio was more than 10. The LLOQs were 2.5 ng/mL and 10 ng/mL for molnupiravir and NHC, respectively. The precision (% RSD) and accuracy (% bias) ranged from -0.41 to 8.76% and -0.16 to 10.32% for analyses of molnupiravir and NHC, respectively (Supplementary Table 1-2). All of the LLOQ values were within ±20%, and those of the other concentrations were within ±15%. These results indicate that our UHPLC–MS/MS analytical method was reproducible, repeatable, and reliable.

The stability data obtained during the analysis of molnupiravir and NHC in rat blood and brain dialysates at low and high concentrations were assessed in triplicate for stability, including bench-top stability, autosampler stability, freeze–thaw stability, and long-term stability, as shown in Supplementary Table 3. The experimental results demonstrated that the RSDs of molnupiravir and NHC were within ±15%, and the results indicated that the analytes in blood and brain dialysates were

stable under these conditions.

The in vitro recoveries of molnupiravir and NHC were assessed using low, medium, and high concentrations of molnupiravir (100, 250, and 500 ng/mL) and NHC (500, 1000, and 2000 ng/mL). The recoveries of molnupiravir and NHC were 12.73 ± 0.09% and 11.47 ± 0.30% for blood probes and 7.58 ± 0.20% and 7.16 ± 0.03% for brain probes (n=3). These results indicated that the recovery depends on the length of the semipermeable membrane and not on the ambient concentration (Supplementary Table 4). The analytical results were corrected using these recovery values by dividing by 0.12 and 0.07 for molnupiravir in blood and brain tissue, respectively, and 0.11 and 0.07 for NHC in blood and brain tissue, respectively.

The average matrix effect values for molnupiravir and NHC in blank dialysate were 94.28-96.15% and 55.44-58.93%, respectively, and these values are presented in Supplementary Table 5. The matrix effect data showed the effect of interfering ions on the analytical target signal. This result revealed that a matrix effect was found for NHC, which was collected for the concentration calculation, and molnupiravir was not significantly affected by the matrix within the analytical range.

(A)

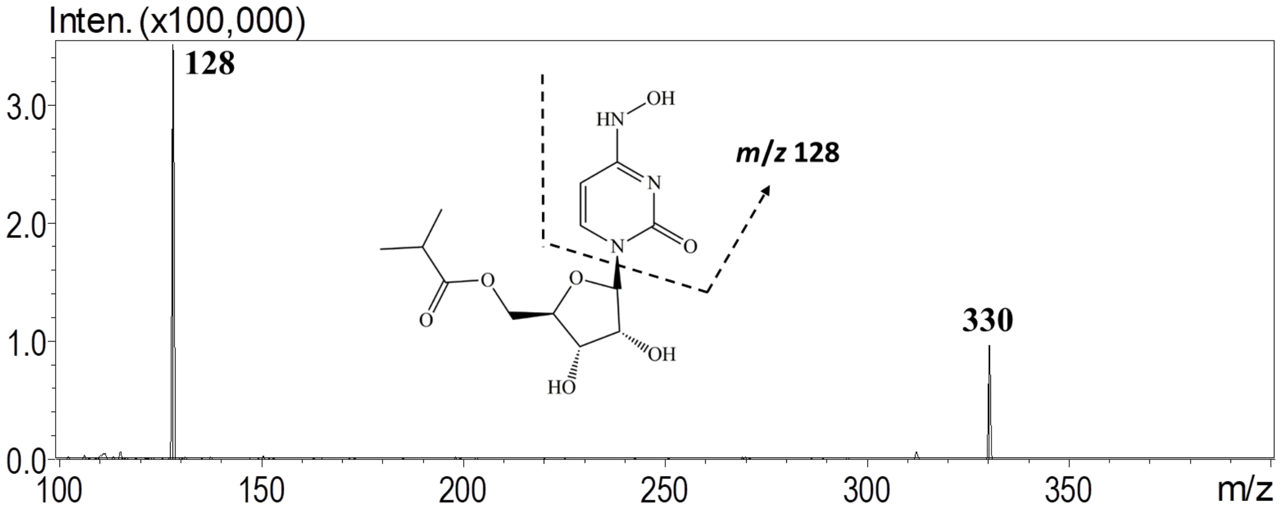

(B)

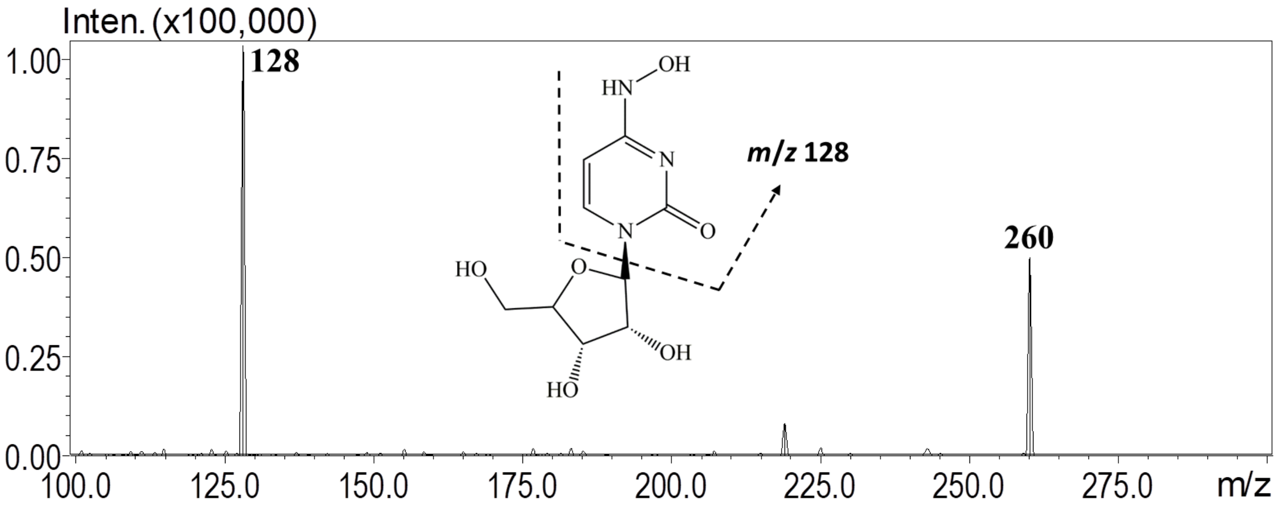

Supplementary Figure 1. MRM product ion mass spectra of (A) molnupiravir at *m/z* 330.2→128.2 and (B) β-*d*-N4-hydroxycytidine at *m/z* 260.1→128.1.

(A)

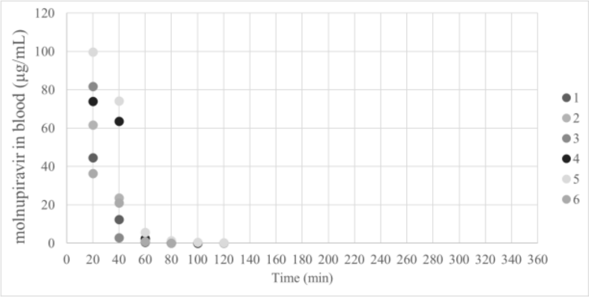

(B)

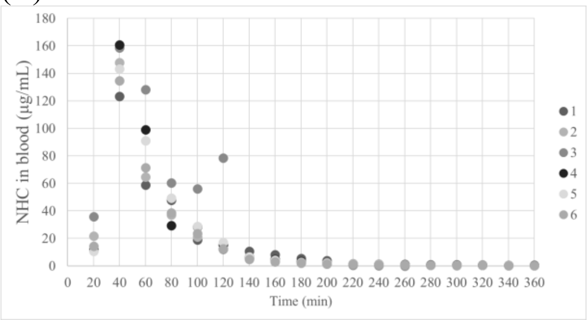

(C)

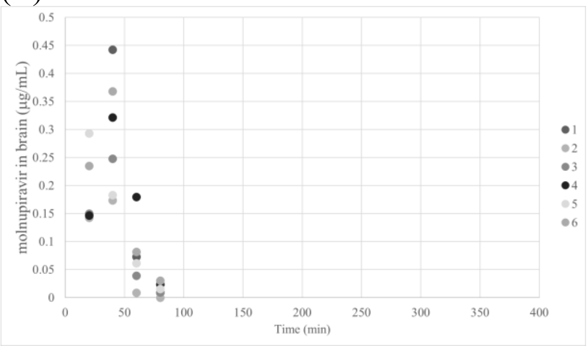

(D)

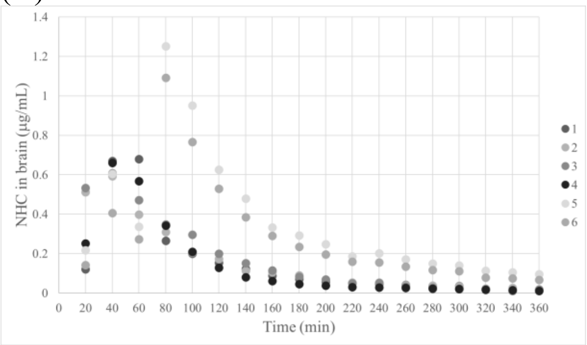

Supplementary Figure 2. The figure showed the raw data that presented in Figure 2. (A) is molnupiravir, (B) is NHC in blood, and (C) is molnupiravir, (D) is NHC in brain after administered molnupiravir (100 mg/kg, i.v.) in male rat.

(A)

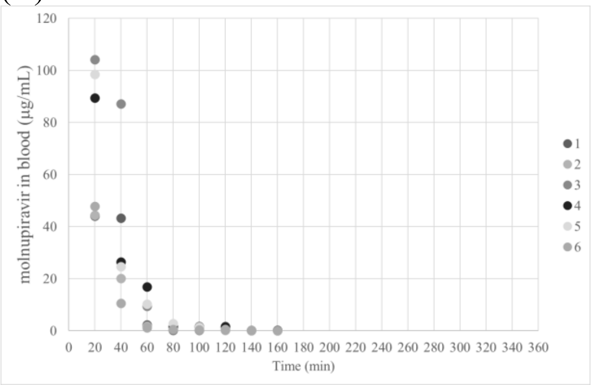

(B)

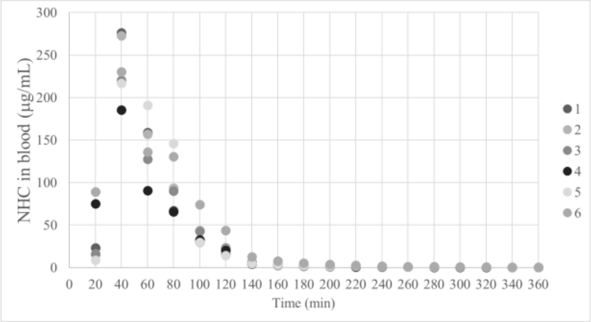

(C)

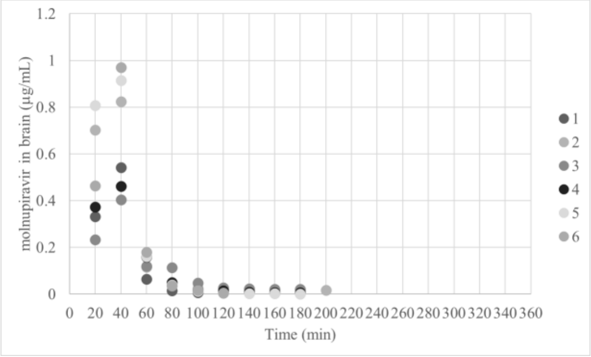

(D)

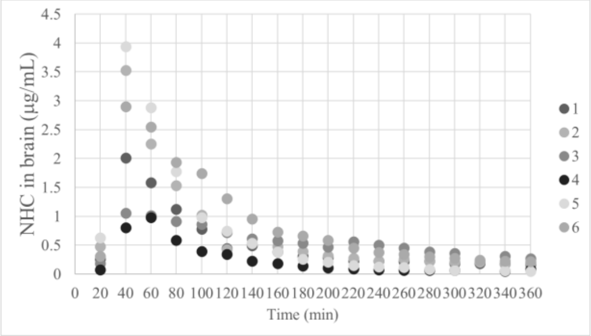

Supplementary Figure 3. The figure showed the raw data that presented in Figure 2. (A) is molnupiravir, (B) is NHC in blood, and (C) is molnupiravir, (D) is NHC in brain after concomitant administered molnupiravir (100 mg/kg, i.v.) and NBMPR (10 mg/kg, i.v.) in male rat.

Supplementary Table 1. Intra- and interday precision (% RSD) and accuracy (% Bias) of the UHPLC–MS/MS method for the determination of molnupiravir in rat blood and brain dialysates

Nominal concentration (ng/mL)	Intraday			Interday		
	Observed concentration (ng/mL)	Precision (% RSD)	Accuracy (% Bias)	Observed concentration (ng/mL)	Precision (% RSD)	Accuracy (% Bias)
Blood						
2.5	2.67 ± 0.12	6.81	4.54	2.57 ± 0.34	13.26	2.92
5	5.44 ± 0.40	8.85	7.35	5.16 ± 0.13	2.52	3.20
25	24.86 ± 0.72	-0.56	2.88	23.54 ± 0.34	1.44	-5.84
250	251.8 ± 2.20	0.73	0.87	250.0 ± 1.14	0.46	0.02
Brain						
2.5	2.27 ± 0.12	-9.24	5.41	2.34 ± 0.31	13.55	-6.35
5	5.53 ± 0.68	10.66	12.44	5.09 ± 0.34	6.76	1.80
25	23.97 ± 0.37	-4.12	1.54	26.22 ± 2.21	8.50	4.86
250	254.0 ± 4.18	1.60	1.65	251.28 ± 1.56	0.62	0.51

Data are expressed as the mean ± SD (n = 5).

Supplementary Table 2. Intra- and interday precision (% RSD) and accuracy (% Bias) of the UHPLC–MS/MS method for the determination of NHC in rat blood and brain dialysates

Nominal concentration (ng/mL)	Intraday			Interday		
	Observed concentration (ng/mL)	Precision (% RSD)	Accuracy (% Bias)	Observed concentration (ng/mL)	Precision (% RSD)	Accuracy (% Bias)
Blood						
10	11.89 ± 0.75	6.31	18.88	10.27 ± 1.22	11.49	2.75
25	23.15 ± 0.97	4.20	-7.39	25.41 ± 1.55	11.39	1.64
100	99.73 ± 1.37	1.37	0.27	103.85 ± 3.76	5.71	3.85
1000	1004 ± 8.55	0.85	0.48	1004 ± 5.69	0.99	0.50
Brain						
10	10.54 ± 1.00	9.60	5.43	10.45 ± 1.16	11.27	4.52
25	23.63 ± 1.53	6.50	-5.48	24.35 ± 2.88	12.00	-2.61
100	96.93 ± 4.10	4.24	-3.07	102.5 ± 5.93	5.79	2.57
1000	993.4 ± 12.56	1.26	-0.65	1004 ± 9.98	0.99	0.44

Data are expressed as the mean ± SD (n = 5).

Supplementary Table 3. Stability of molnupiravir and NHC in rat blood and brain dialysates

Nominal concentration (ng/mL)	Autosampler stability (%)	Short-term stability (%)	Freeze–thaw stability (%)	Long-term stability (%)
Molnupiravir				
Blood				
5	101.11 ± 1.81	104.95 ± 0.92	105.51 ± 0.54	107.88 ± 0.59
250	100.33 ± 1.00	101.21 ± 3.94	104.37 ± 5.11	97.50 ± 2.58
Brain				
5	98.90 ± 0.25	100.50 ± 1.33	99.43 ± 1.12	98.15 ± 1.89
250	101.22 ± 0.20	104.89 ± 1.54	100.88 ± 5.12	98.54 ± 0.65
NHC				
Blood				
25	98.75 ± 2.94	101.78 ± 6.84	98.21 ± 8.39	101.56 ± 4.45
1000	99.63 ± 0.14	100.91 ± 0.94	100.71 ± 0.76	100.11 ± 0.66
Brain				
25	101.44 ± 3.19	101.17 ± 1.49	93.78 ± 4.13	108.86 ± 3.06
1000	101.64 ± 1.20	100.89 ± 0.64	101.17 ± 0.33	100.97 ± 0.83

Data are expressed as the mean ± SD (n = 3).

Supplementary Table 4. In vitro microdialysis recovery (%) of molnupiravir and NHC using ACD solution as the perfusate solution. Data are expressed as the mean ± SEM (n=3).

Concentration (ng/mL)	Recovery (%) in blood probe	Recovery (%) in brain probe
Molnupiravir		
100	12.83 ± 0.20	7.77 ± 0.07
250	12.62 ± 0.16	7.31 ± 0.06
500	12.73 ± 0.17	7.67 ± 0.13
average	12.73 ± 0.09	7.58 ± 0.20
β-d-N4-hydroxycytidine		
500	11.90 ± 0.12	7.14 ± 0.11
1000	11.27 ± 0.07	7.21 ± 0.13
2000	11.25 ± 0.06	7.14 ± 0.04
average	11.47 ± 0.30	7.16 ± 0.03

The probe in vitro recovery was determined by comparing the concentration of input (C_{in}) and output (C_{out}) solution of the analyte according to the equation of recovery (%) = (C_{out} / C_{in}) × 100%.

Supplementary Table 5. The matrix effects of molnupiravir and NHC in rat blood and brain dialysates

Nominal concentration (ng/mL)	Blood	Brain
Molnupiravir		
5	94.28 ± 1.69	94.89 ± 6.57
250	95.11 ± 0.52	96.15 ± 0.63
NHC		
25	55.44 ± 2.66	58.55 ± 2.50
1000	55.86 ± 0.40	58.93 ± 0.36

Data are expressed as the ratio (%) of the mean peak area of a dialysate spiked with the standard solution to the mean peak area of the standard solution prepared in ACD solution and are as expressed the mean ± SD (n = 3).
