# Supplementary material for: Transporter modulation of molnupiravir and its metabolite β-D-N4-hydroxycytidine across the blood-brain barrier in a rat
Source: Commun Med (Lond). 2023 Oct 19;3:150. doi: 10.1038/s43856-023-00383-w (PMC10587300; doi:10.1038/s43856-023-00383-w)
Supplement: Supplementary file 6 — Description of Additional Supplementary Files [file 43856_2023_383_MOESM6_ESM.docx]

Description of Additional Supplementary Files

File Name: Supplementary Data 1

Description: This file provides the analytical method validations of Data for Accuracy and Precision, Data for Stability, Data for Matrix effect, Data for Recovery.

File Name: Supplementary Data 2

Description: This file provides the pharmacokinetic data of Data for molnupiravir in blood and brain, Data for NHC in blood and brain, and treatment of NBMPR; Data for molnupiravir in blood and brain; Data for NHC in blood and brain.
